# Supplementary material for: Large transverse thermoelectric effect induced by the mixed-dimensionality of Fermi surfaces
Source: Nat Commun. 2024 May 9;15:3907. doi: 10.1038/s41467-024-48217-0 (PMC11081953; doi:10.1038/s41467-024-48217-0)
Supplement: Supplementary file 1 — Supplementary Information [file 41467_2024_48217_MOESM1_ESM.pdf]

SUPPLEMENTARY INFORMATION

A. Single crystal growth

The single crystal grown using the Czochralski method is shown in Fig. S1**a**. As shown in Fig. S1**b**, the clear Laue spots indicate the high quality of the single crystal. After determining the crystal orientation, the grown single crystals were cut using a spark cutter. We have conducted trasnsverse thermopower measurements on samples No.1 (1.2 mm × 1.5 mm × 0.5 mm) and No.2 (1.5 mm × 1.6 mm × 0.7 mm), which were cut from the Czochralski grown single-crystal ingot.

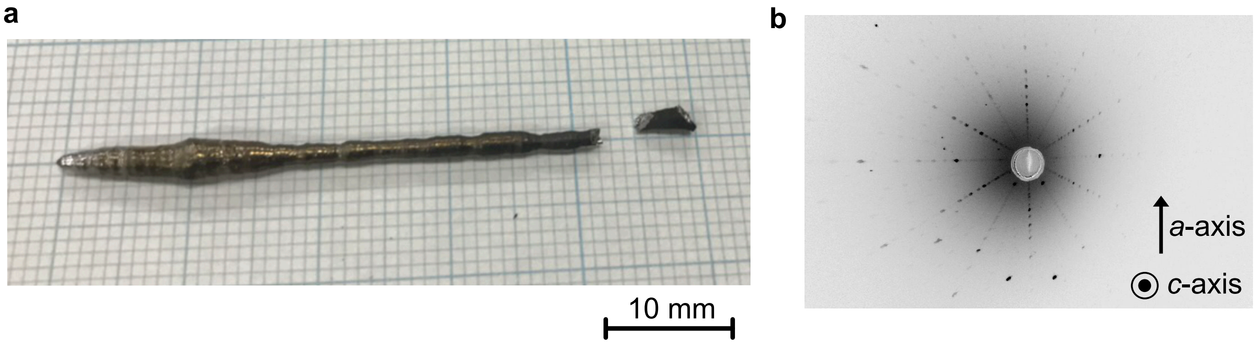

FIG. S1. **Single crystal growth and orientation. a**, Single crystals of $LaPt_2B$ grown using the Czochralski method. **b**, Laue photograph of $LaPt_2B$ single crystal.

B. Band structure and Fermi surfaces

Figures S2**a** and S2**b** show the calculated band structures and the density of states (DOS) in the absence and presence of spin-orbit coupling (SOC), respectively. Since $LaPt_2B$ has a chiral crystal structure, the energy bands are split owing to the inclusion of SOC except at the time-reversal-invariant momenta (TRIMs) [1]. Thus, the Kramers-Weyl points may emerge at TRIMs and the associated nontrivial transport phenomena in magnetic fields are of great interests, while we here focus on the transverse thermoelectricity induced by the goniopolar conduction in zero magnetic field. The electronic states near the Fermi energy E_F are composed mostly of La 5d, B 2p, Pt 6p, and Pt 5d orbitals, which is qualitatively consistent with the previous study on the related ternary compounds [2]. Note that other orbital contributions are negligibly small near E_F and not shown in Figs. S2**a** and S2**b**.

The Fermi surfaces obtained in the scalar and full relativistic calculations are shown in Fig. S3. The number of Fermi surfaces is doubled in the relativistic calculations owing to lack of the inversion symmetry and the overall shapes of the Fermi surfaces are essentially same to those for the scalar relativistic calculations. Indeed, the calculated transport coefficients with including SOC are qualitatively similar to those without SOC as shown later.

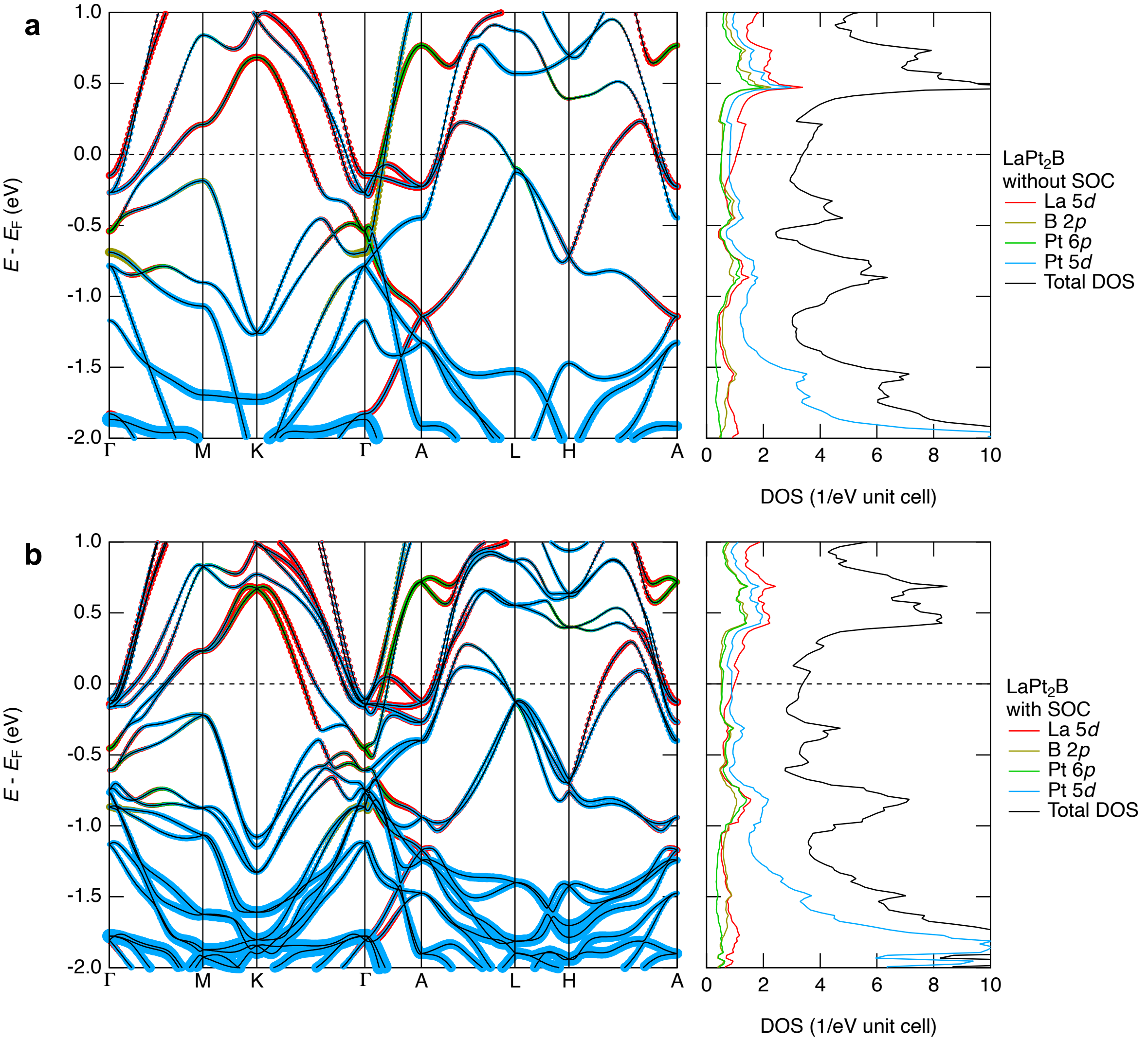

FIG. S2. **Electronic band structures and DOS.** The first-principles calculations were performed in the absence (**a**) and presence (**b**) of SOC.

C. Band-resolved anisotropic transport coefficients

We calculated the band-resolved partial electrical conductivity σ_{ii}^{n} and the partial Peltier conductivity α_{ii}^{n} $(= \sigma_{ii}^{n} S_{ii}^{n})$ ($i = a, c$ and $n = \alpha, \beta, \gamma, \delta, \varepsilon$) (Figs. S3**a** and S3**b**). In this calculations, the relaxation time τ was set to the constant of $\tau = 10^{-14}$ s and the temperature was set to 300 K. The SOC is not included in the calculations. Near the Fermi energy ($\mu = E_{\mathrm{F}}$), band-dependent transport anisotropy is clearly observed as expected in the shape of the Fermi surfaces shown in Fig. S3**c**. In addition, the polarity of carriers for each band is found in the partial Peltier conductivity α_{ii}^{n}: At $\mu = E_{\mathrm{F}}$, the partial Peltier conductivity is positive for both directions in the hole-like α

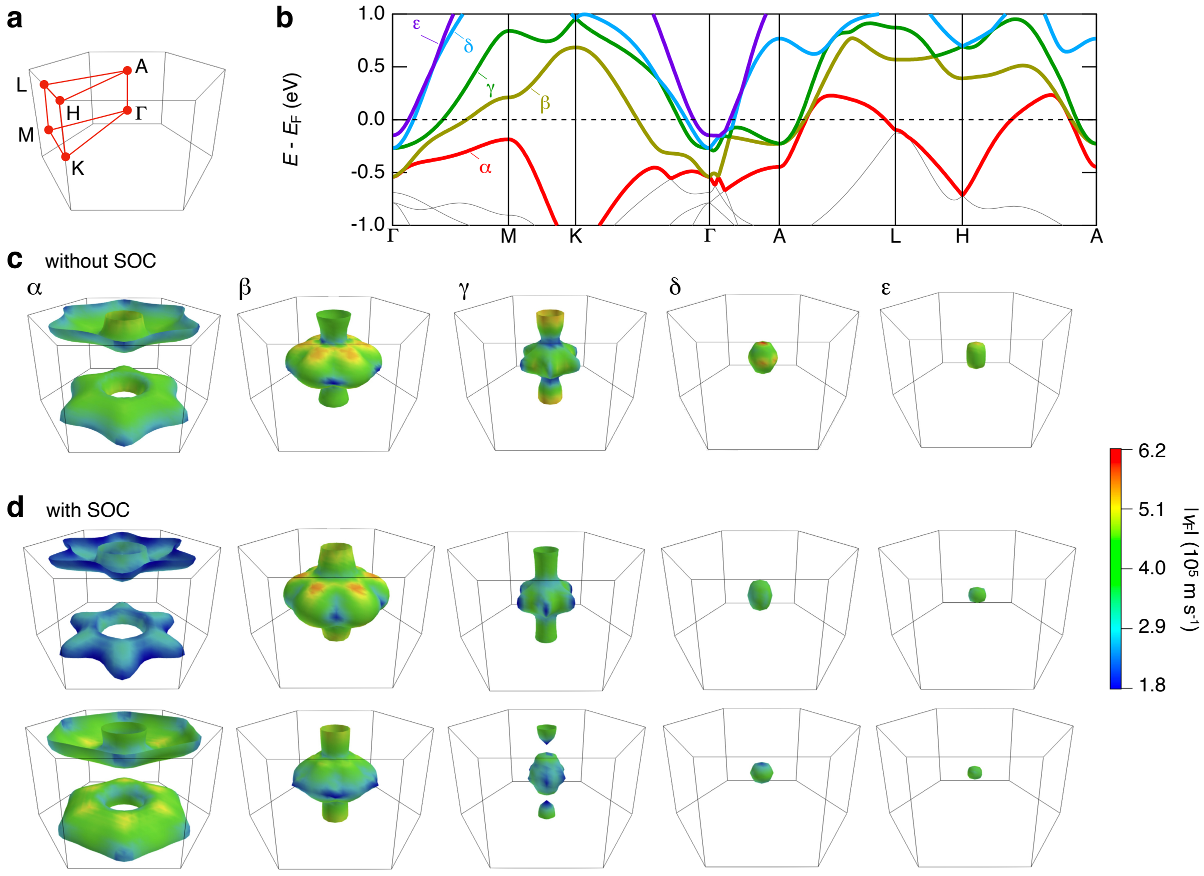

FIG. S3. **Fermi surfaces of $LaPt_2B$. a**, Brillouin zone and high symmetry points in the hexagonal lattice. **b**, Calculated band structure near the Fermi energy E_F in the absence of SOC. Five bands crossing E_F are labelled as α, β, γ, δ, and ε, and corresponding Fermi surfaces are shown in **c**. Fermi surfaces are drawn using FermiSurfer program [3]. The color scale indicates the magnitude of the Fermi velocity v_{F}. **d**, Fermi surfaces obtained in the fully relativistic calculations with SOC.

sheet. Other electron-like bands exhibit negative Peltier conductivity at $\mu = E_F$.

Transport anisotropy is then evaluated as the ratio of the electrical conductivity $\sigma^n_{cc}/\sigma^n_{aa}$ (Fig. S4**c**) and the ratio of the Peltier conductivity $\alpha^n_{cc}/\alpha^n_{aa}$ (Fig. S4**d**) at $T = 300$ K. Note that an isotropic three-dimensional transport may be expected when these anisotropy ratios are unity. The anisotropy of the hole-like α sheet is $\sigma^\alpha_{cc}/\sigma^\alpha_{aa} = 2.46 > 1$ and $\alpha^\alpha_{cc}/\alpha^\alpha_{aa} = 3.03 > 1$, indicating that the hole conduction with positive thermopower is preferred along the c-axis direction. On the other hand, electron-like γ sheet has a small ratio of $\sigma^\gamma_{cc}/\sigma^\gamma_{aa} = 0.43 < 1$ and $\alpha^\alpha_{cc}/\alpha^\alpha_{aa} = 0.13 < 1$, to drive the electron conduction with negative thermopower along the in-plane direction, reflecting the cylindrical shape of the γ sheet (Fig. S3**c**). Note that, however, electron-like β sheet has relatively large anisotropy values of $\sigma^\beta_{cc}/\sigma^\beta_{aa} = 2.18 > 1$ and $\alpha^\beta_{cc}/\alpha^\beta_{aa} = 1.97 > 1$, since the expanded area of the β sheet around Γ point has relatively high velocity along the c axis.

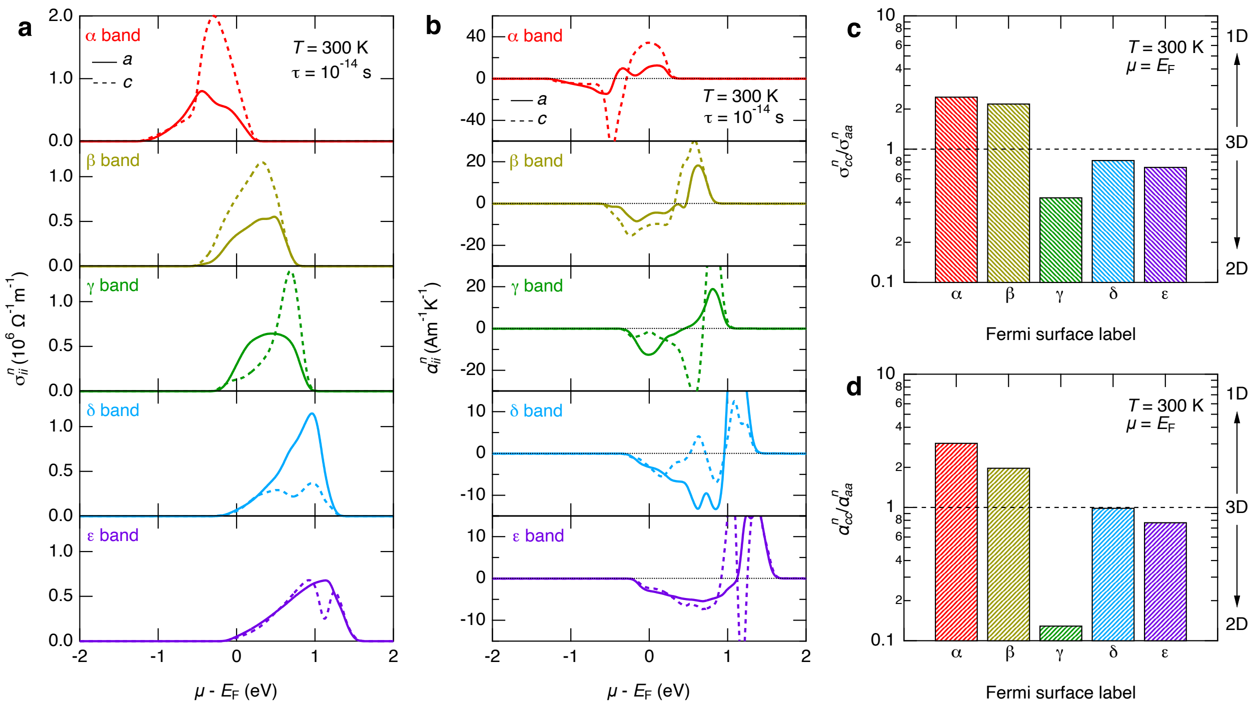

FIG. S4. **Band-resolved transport properties and anisotropy.** **a**,**b**, Calculated electrical conductivity σ^n_{ii} (**a**) and the Peltier conductivity α^n_{ii} $(= \sigma^n_{ii}S^n_{ii})$ (**b**) of the n band ($n = \alpha, \beta, \gamma, \delta, \varepsilon$) along the i direction ($i = a, c$) as a function of chemical potential μ. The solid and dashed curves represent the in-plane ($\sigma^n_{aa}, \alpha^n_{aa}$) and out-of-plane ($\sigma^n_{cc}, \alpha^n_{cc}$) components, respectively. **c**,**d**, Transport anisotropy of the n band defined as $\sigma^n_{cc}/\sigma^n_{aa}$ (**c**) and $\alpha^n_{cc}/\alpha^n_{aa}$ (**d**) at $T = 300$ K.

D. Transport coefficients calculated with SOC

Figures S5**a** and S5**b** show the calculated thermopower along the in-plane (S_{aa}) and out-of-plane (S_{cc}) directions. In the scalar relativistic calculations without SOC, we used Boltztrap [4] and Boltzwann [5] codes, the results of which coincide with each other (solid symbols in Figs. S5**a** and S5**b**). We used projections of La $5d$, B $2p$, Pt $6p$, and Pt $5d$ orbitals to construct the maximally-localized Wannier functions and the dense k-mesh grid of $100 \times 100 \times 100$ was used for the calculation of the transport coefficients in the Boltzwann module. In the full relativistic calculations with SOC, we only used Boltzwann module since the SOC calculations were not supported in the Boltztrap code. Although the calculation results with SOC (open symbols in Figs. S5**a** and S5**b**) are slightly different from those without SOC, the overall behavior of the goniopolar conduction is well reproduced.

E. Orbital weights on the Fermi surfaces

Figure S6**a** shows the orbital-projected Fermi surfaces for La, Pt, and B. As seen in the DOS (Fig. S2), the dominant contributions near the Fermi energy arise from La $5d$, B $2p$, Pt $6p$ and Pt $5d$. The Pt orbitals have a large weight in the α sheet, and the B orbital has a relatively large contribution in the same position as the Pt orbitals due to Pt-B hybridization. La and Pt orbitals

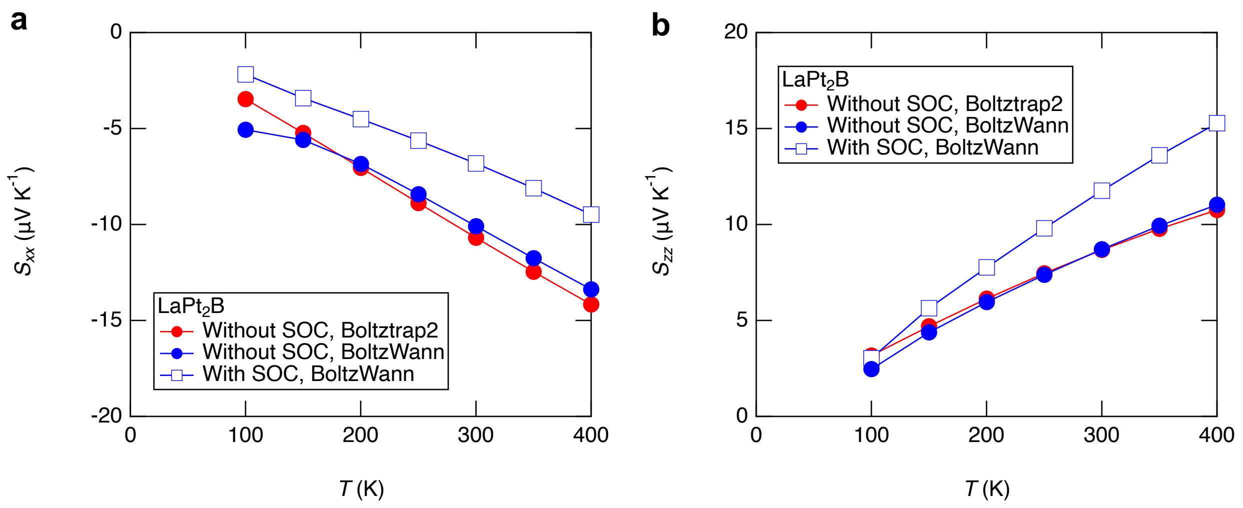

FIG. S5. **Calculated thermopower of $LaPt_2B$.** Calculated thermopower for the in-plane (**a**) and the out-of-plane directions (**b**).

have large contributions in the quasi-2D Fermi surface (γ sheets).

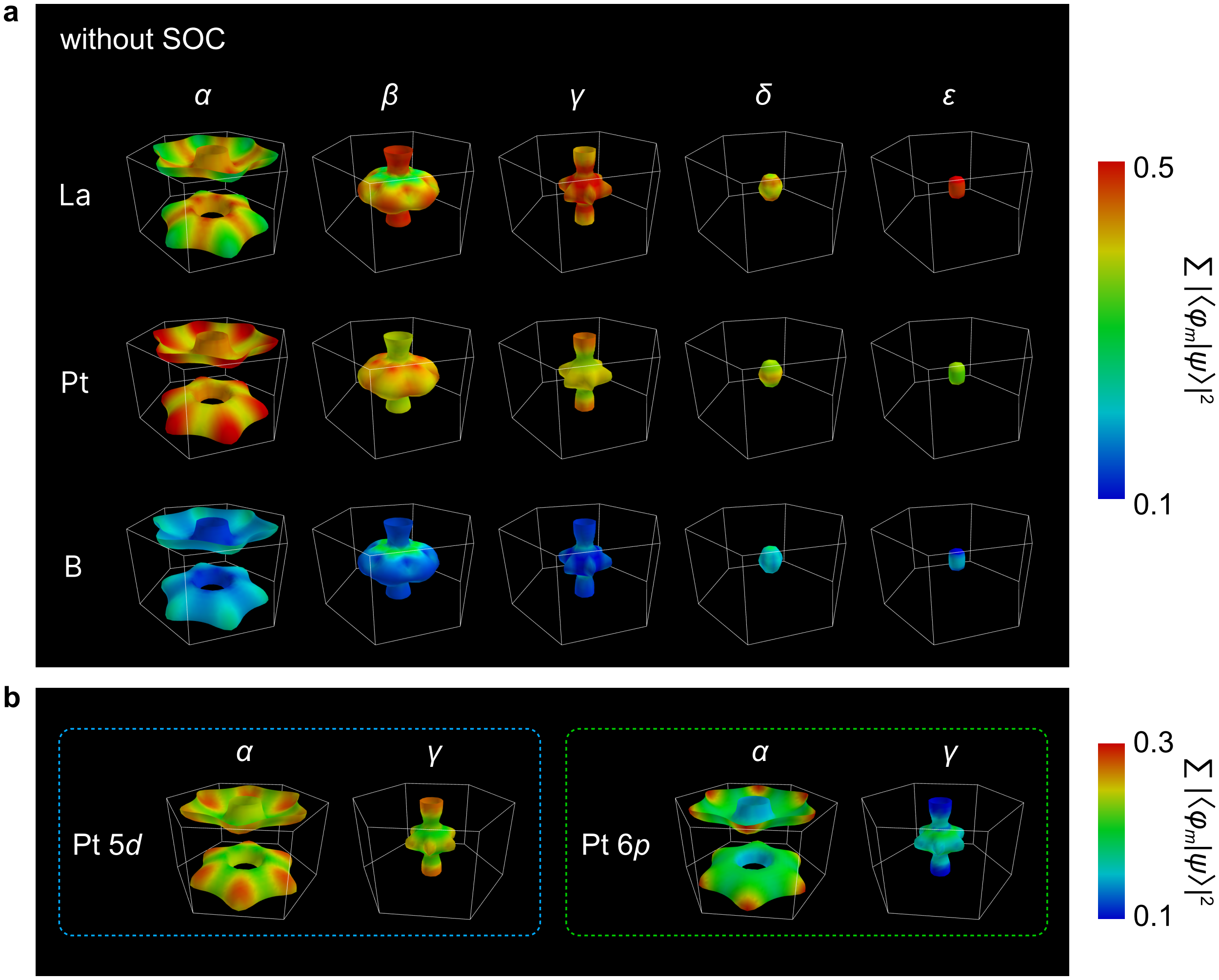

FIG. S6. **Orbital weights on the Fermi surfaces.** **a**, Orbital weights for La, Pt, and B orbitals to n sheets (n = α, β, γ, δ, ε). The color scale indicates the sum of the projections of the m orbitals for each atom. **b**, Orbital weights for Pt 5d and Pt 6p orbitals to α and γ sheets.

The contributions to the α and γ sheets decomposed into Pt-5d and Pt-6p contributions are shown in Fig. S6**b**. In the α sheet, the Pt 5d orbitals have a large contribution in the out-of-plane direction, while the Pt 6p orbitals have a large contribution in a restricted region in the in-plane direction. In the γ sheet, the Pt 5d electrons contribute significantly to the cylindrical region near the $k_z = \pm 1/2$ (A-L-H) plane.

As mentioned above, the characteristic crystal structure of $LaPt_2B$ may be closely related to the dimensionality of the Fermi surface responsible for goniopolar conduction. In the quasi-one-

dimensional hole Fermi surface (α sheet), the hybridization of Pt and B plays an important role, while in the two-dimensional electron Fermi surface (γ sheets), both La and Pt appear to contribute significantly.

F. Transverse thermopower measurement

We simultaneously measured the voltage drops ΔV and the temperature difference ΔT along the transverse and longitudinal directions using four thermocouples. Here we show results of the transverse thermopower measurements of a constantan sheet as a reference sample. Figures S7**a** and S7**b** show the temperature dependence of ΔT and ΔV for both longitudinal (xx) and transverse (yy) directions. For the longitudinal direction, a significant ΔT and a corresponding ΔV are

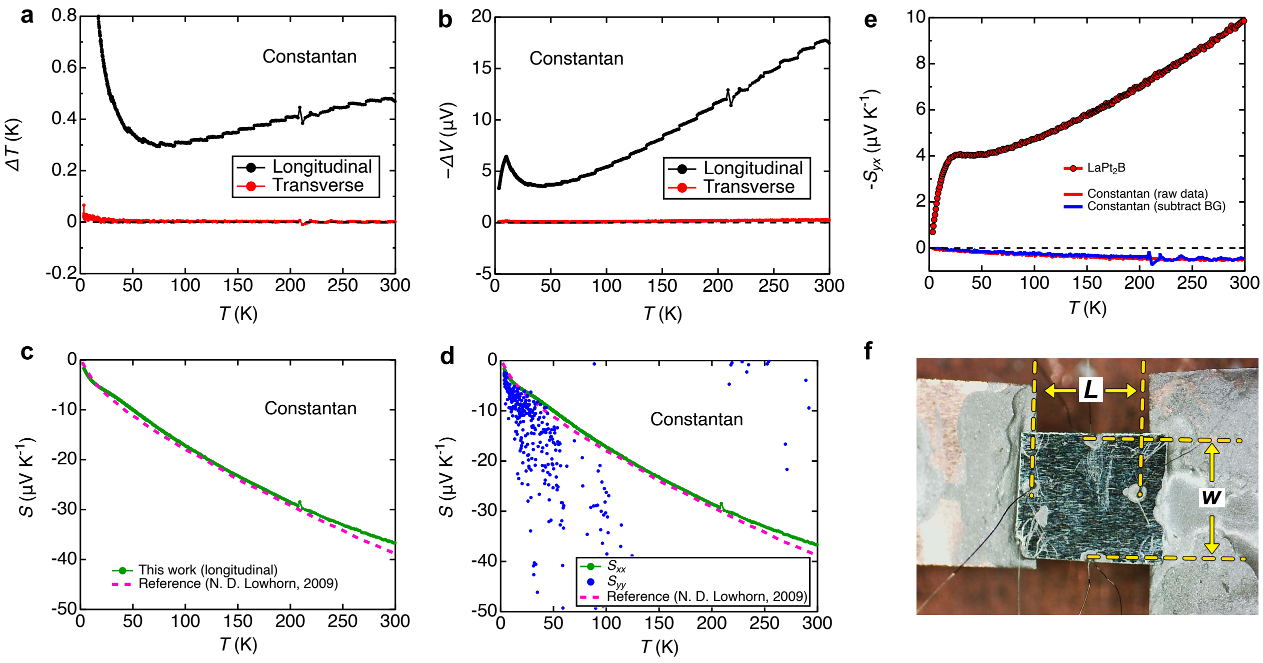

FIG. S7. **Transverse thermopower measurement of a reference sample (constantan sheet).** **a,b**, Temperature dependence of ΔT (**a**) and ΔV (**b**) for longitudinal (xx) and transverse (yy) directions. **c**, Longitudinal (ordinary) Seebeck coefficient of a constantan sheet with the reported values in Ref. [6]. **d**, Comparison of S_{xx} and S_{yy}. **e**, Negligible small transverse thermopower $S_{yx} = (\Delta V_y/w)/(\Delta T_x/L)$ of a constantan sheet with the giant transverse thermopower of $LaPt_2B$. **f**, Experimental setup for transverse thermopower measurement of a constantan sheet.

observed. On the other hand, both ΔT and ΔV are of negligible magnitude for the transverse direction. These results are in contrast to the results for the goniopolar conductor $LaPt_2B$ (Figs. 3**b** and 3**c**, main text). As shown in Fig. S7**c**, $S_{xx} = \Delta V_x/\Delta T_x$ shows good agreement with the reported value[6] for the longitudinal direction. However, $S_{yy} = \Delta V_y/\Delta T_y$ cannot be measured due to the negligible small ΔT_y and ΔV_y for the transverse direction, as shown in Fig. S7**d**. The experimentally observed transverse thermopower, calculated from the distinct ΔT for the longitudinal direction and the negligible ΔV for the transverse direction, is found to be extremely small. In principle, S_{yx} is negligible in conventional materials. Figure S7**f** shows the experimental setup using four thermocouples.

Figure S8**a** shows the temperature dependence of transverse thermopower S_{yx} and longitudinal thermopower S_{xx} of $LaPt_2B$. The observed $S_{yx}(T)$ and $S_{xx}(T)$ can be well explained by equations of $S_{yx} = (S_{aa} - S_{cc})\cos\phi\sin\phi$ and $S_{xx} = S_{aa}\cos^2\phi + S_{cc}\sin^2\phi$, where S_{aa} and S_{cc} are the thermopower for a- and c-axes. Figures S8**b** and S8**c** show calculated S_{yx} and S_{xx} for several angles. As shown in Figs. S8**b** and S8**c**, the experimental S_{yx} and S_{xx} data indicate that the direction of the temperature gradient is approximately 45 degrees from a- and c-axes.

We have investigated sample dependence of the longitudinal and transverse thermoelectric power of $LaPt_2B$, as shown in Figs. S9**a** and S9**b**. The sample dependence of S_{xx} and S_{yx} is attributed to differences in sample quality, discrepancies in the estimation of sample dimension,

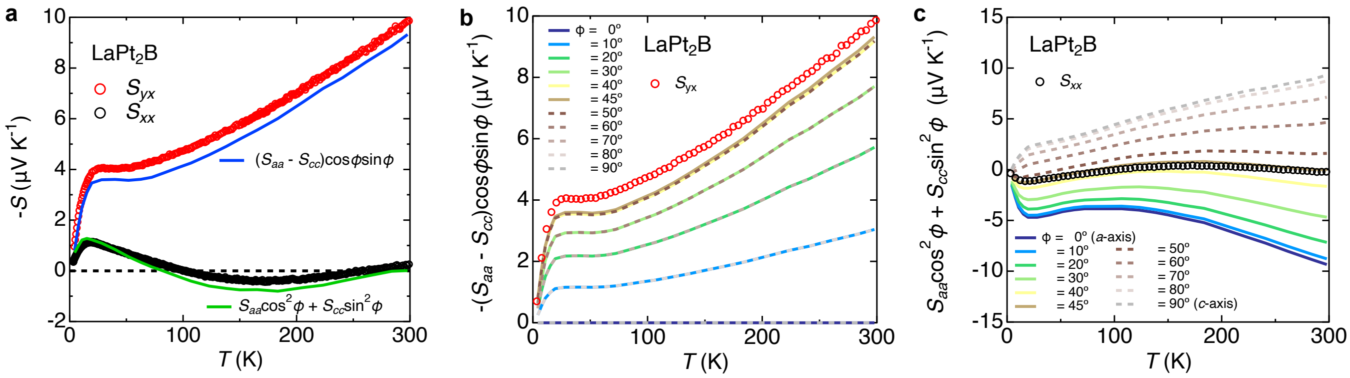

FIG. S8. **Temperature dependence of transverse and longitudinal thermopower. a**, Temperature dependence of transverse thermopower S_{yx} and longitudinal thermopower S_{xx}. Solid lines are calculated transverse thermopower $S_{yx} = (S_{aa} - S_{cc})\cos\phi\sin\phi$ and calculated longitudinal thermopower $S_{xx} = S_{aa}\cos^2\phi + S_{cc}\sin^2\phi$ for $\phi = 45°$. **b,c**, Comparison of experimental and calculated transverse thermopower S_{yx} (**b**) and longitudinal thermopower S_{xx} (**c**) for several angles ϕ.

and experimental setup errors. However, the giant transverse thermopower has been observed in several $LaPt_2B$ single crystals, as shown in Figs. S9**a**.

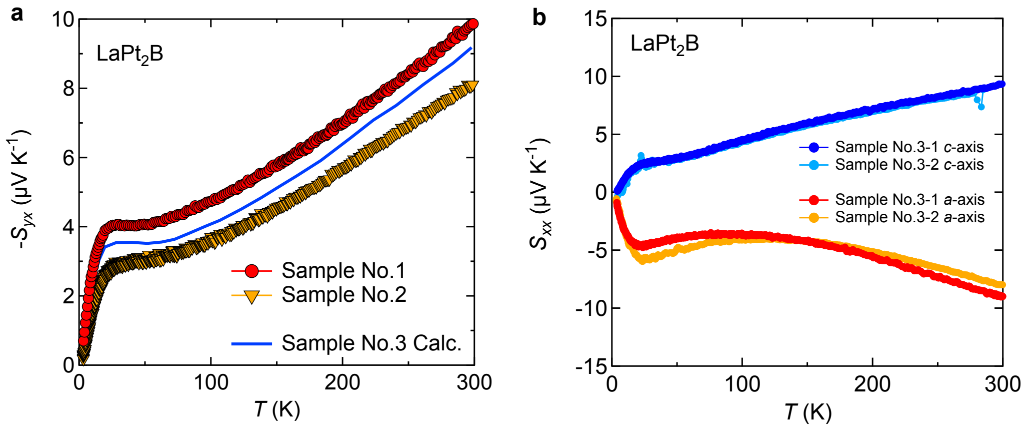

FIG. S9. **Sample dependence of transverse and longitudinal thermopower. a,b**, Sample dependence of transverse thermopower (**a**) and (longitudinal) Seebeck coefficient (**b**). The solid line in panel **a** represents the transverse thermopower calculated from S_{aa} and S_{cc} of sample No.3 based in Eq.(9).

G. Thermal conductivity

Figure S10**a** shows the temperature dependence of thermal conductivity. The thermal conductivity was measured by a steady-state method. The temperature gradient was applied using a 2.7 kΩ chip resistor and detected using manganin-constantan thermocouples. All connections were made using silver paste (Dupont 4922). As shown in Figs. S10**b** and S10**c**, both thermal conductivity and Seebeck coefficient exhibit a peak structure attributed to the phonon drag effect at approximately 20 K.

H. Hall effect

In the Hall measurements, the Hall resistance ($R_{yx} = V_y/I_x$) were measured using a delta mode technique (Keithley 2182A/6220). We defined the electrical current direction as x and the magnetic field direction as z. To cancel out misalignment contributions, we measured the genuine Hall resistance as $R_{yx} = [R_{yx}^{\mathrm{meas}}(+H) - R_{yx}^{\mathrm{meas}}(-H)]/2$. Figure S11**a** shows the field dependence of Hall resistivity ρ_{yx} of $LaPt_2B$ for $J \parallel a$ and $J \parallel c$. In contrast to the clear goniopolar conductivity

observed in the Seebeck coefficient, the Hall coefficient was negative for both terminal configurations. The sign of the Hall coefficient for $J \parallel a$, $E \parallel b$ (b is the in-plane direction perpendicular to the a-axis and c-axis), $H \parallel c$ is consistent with the results of Seebeck coefficient for in-plane direction. On the other hand, the sign of R_H for $J \parallel c$, $E \parallel b$, $H \parallel a$ is reversed compared to the sign of the Seebeck coefficient for the c-axis. This sign reversal is attributed to the influence of the multi-carrier effect, as the R_H is evaluated in the cb-plane (c and b direction are the out-of-plane and in-plane directions, respectively). Figure S11**a** shows the Hall coefficient R_H calculated using the BoltzTraP2 code for $J \parallel a$ and $J \parallel c$. The experimentally obtained sign and magnitude of R_H are in good agreement with those of calculated R_H. The experimental and calculated R_H are almost temperature independent.

I. Analysis of transverse Peltier conductivity and Peltier angle for transverse thermoelectric systems

The Peltier conductivity tensor $\hat{\alpha}$ is expressed as

$$\hat{\alpha} = \hat{\sigma}\hat{S} = \begin{pmatrix} \sigma_{xx} & \sigma_{xy} \\ \sigma_{yx} & \sigma_{yy} \end{pmatrix} \begin{pmatrix} S_{xx} & S_{xy} \\ S_{yx} & S_{yy} \end{pmatrix}, \tag{1}$$

where $\hat{\sigma}$ and $\hat{S}$ are the electrical conductivity tensor and the thermopower tensor. The off-diagonal term (transverse Peltier conductivity) to induce the transverse current $J_y = \alpha_{yx}(-\nabla_x T)$ is then given

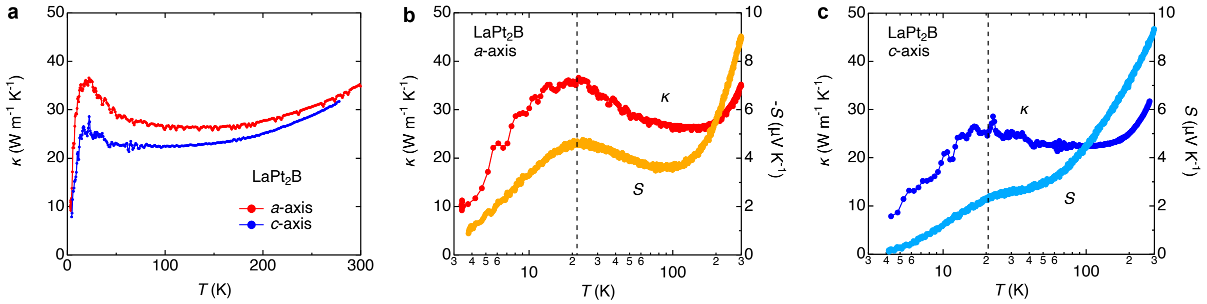

FIG. S10. **Thermal conductivity of LaPt$_2$B. a**, Temperature dependence of thermal conductivity of LaPt$_2$B. **b,c**, Comparison of temperature dependence of thermal conductivity and Seebeck coefficient for a-axis (**b**) and c-axis (**c**). Both thermal conductivity and Seebeck coefficient show a peak structure at approximately 20 K.

as

$$\alpha_{yx} = \sigma_{yx} S_{xx} + \sigma_{yy} S_{yx}. \tag{2}$$

The crystallographic ac coordinate is rotated with the angle ϕ from the xy coordinate. In the xy coordinate, the thermopower is given as

$$\hat{S} = \hat{R}_\phi \hat{S}^{(ij)} \hat{R}_\phi^{-1} = \begin{pmatrix} S_{aa}\cos^2\phi + S_{cc}\sin^2\phi & (S_{aa} - S_{cc})\cos\phi\sin\phi \\ (S_{aa} - S_{cc})\cos\phi\sin\phi & S_{aa}\sin^2\phi + S_{cc}\cos^2\phi \end{pmatrix}, \tag{3}$$

where $\hat{S}^{(ij)}$ is the thermopower tensor in the crystallographic ac coordinate and $\hat{R}_\phi$ is a rotational matrix;

$$\hat{S}^{(ij)} = \begin{pmatrix} S_{aa} & 0 \\ 0 & S_{cc} \end{pmatrix}, \quad \hat{R}_\phi = \begin{pmatrix} \cos\phi & -\sin\phi \\ \sin\phi & \cos\phi \end{pmatrix}. \tag{4}$$

For $\phi = 45$ degrees, we obtain

$$\hat{S} = \frac{1}{2}\begin{pmatrix} S_{aa} + S_{cc} & S_{aa} - S_{cc} \\ S_{aa} - S_{cc} & S_{aa} + S_{cc} \end{pmatrix}. \tag{5}$$

Similarly, we obtain the electrical conductivity tensor as

$$\hat{\sigma} = \frac{1}{2}\begin{pmatrix} \sigma_{aa} + \sigma_{cc} & \sigma_{aa} - \sigma_{cc} \\ \sigma_{aa} - \sigma_{cc} & \sigma_{aa} + \sigma_{cc} \end{pmatrix}. \tag{6}$$

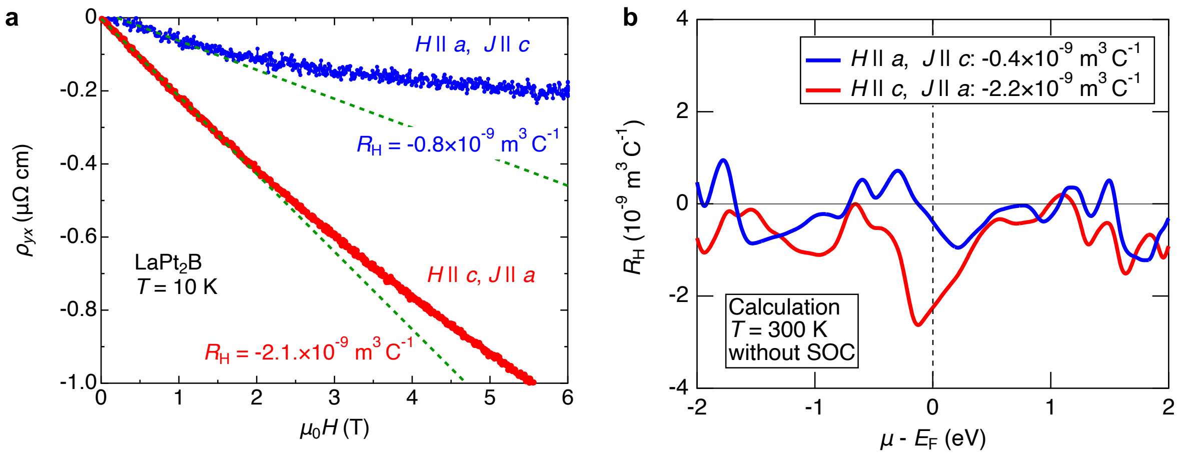

FIG. S11. **Hall effect of $LaPt_2B$. a**, Field dependence of Hall resistivity of $LaPt_2B$ for $H \parallel a$ and $J \parallel c$ (blue markers) and for $H \parallel c$ and $J \parallel a$ (red markers) at $T = 10$ K. **b**, Calculated Hall coefficient R_H for $H \parallel a$ and $J \parallel c$ (blue solid line) and for $H \parallel c$ and $J \parallel a$ (red solid line). The dashed line indicates the Fermi level.

At zero magnetic field, the off-diagonal components of electrical conductivity (σ_{yx}) become zero when heat flow or electric current is applied along the crystallographic axes. However, by rotating the coordinate axes, the off-diagonal components of electrical conductivity appear even in a zero magnetic field.

We have measured both S_{xx} and S_{yx} of $LaPt_2B$. We evaluated the Peltier conductivity using the experimentally obtained S_{xx} and S_{yx} for $LaPt_2B$ as follows:

$$\alpha_{yx} = \frac{1}{2}(\sigma_{aa} + \sigma_{cc})S_{yx} + \frac{1}{2}(\sigma_{aa} - \sigma_{cc})S_{xx}. \tag{7}$$

Similarly, we evaluated the Peltier angle [7, 8] for $LaPt_2B$ using measured S_{xx} and S_{yx} as

$$\tan\theta_{\mathrm{P}} = \frac{|(\sigma_{aa} + \sigma_{cc})S_{yx} + (\sigma_{aa} - \sigma_{cc})S_{xx}|}{|\sigma_{aa}S_{aa} + \sigma_{cc}S_{cc}|}. \tag{8}$$

For other goniopolar systems, S_{xx} and S_{yx} have not been directly measured, and we evaluated the transverse Peltier conductivity as

$$\alpha_{yx} = \frac{1}{2}(\sigma_{aa}S_{aa} - \sigma_{cc}S_{cc}). \tag{9}$$

The Peltier angle for other goniopolar systems were evaluated from $\mathbf{J} = \hat{\alpha}(-\nabla T)$ as

$$\tan\theta_{\mathrm{P}} = \frac{|\alpha_{yx}|}{\sqrt{\alpha_{xx}\alpha_{yy}}} = \frac{|\alpha_{aa} - \alpha_{cc}|}{|\alpha_{aa} + \alpha_{cc}|} \tag{10}$$

$$= \frac{|\sigma_{aa}S_{aa} - \sigma_{cc}S_{cc}|}{|\sigma_{aa}S_{aa} + \sigma_{cc}S_{cc}|}. \tag{11}$$

Here, we consider the validity of the evaluation of the Peltier conductivity in $LaPt_2B$. Figure S12 shows the comparison of α_{yx} in $LaPt_2B$ evaluated by several expressions. The α_{yx} estimated from Eqs. (7) and (9) yields approximately the same value, as shown in Fig. S12. We can also evaluate the Peltier conductivity using Eq. (2) by directly measuring σ_{yy} and σ_{yx}. Note that measuring the off-diagonal components (σ_{xy} and σ_{yx}) is very challenging when rotating the current direction from the crystallographic axes due to the small absolute value and the issues related to terminal misalignment. In addition, the term $\sigma_{yx}S_{xx}$ in the transverse Peltier conductivity becomes very small due to both the small σ_{yx} and S_{xx} in an isotropic goniopolar conductor. As shown in Fig. S12, the Peltier conductivity evaluated by $\alpha_{yx} = \sigma_{yx}S_{xx} + \sigma_{yy}S_{yx} \sim \sigma_{yy}S_{yx}$ exhibits a similar behavior to the Peltier conductivity evaluated by other expressions.

The Peltier angle has a slightly different physical interpretation compared to the commonly discussed Nernst angle. The Nernst angle θ_{N} is the angle between the electric field $\boldsymbol{E}$ and the

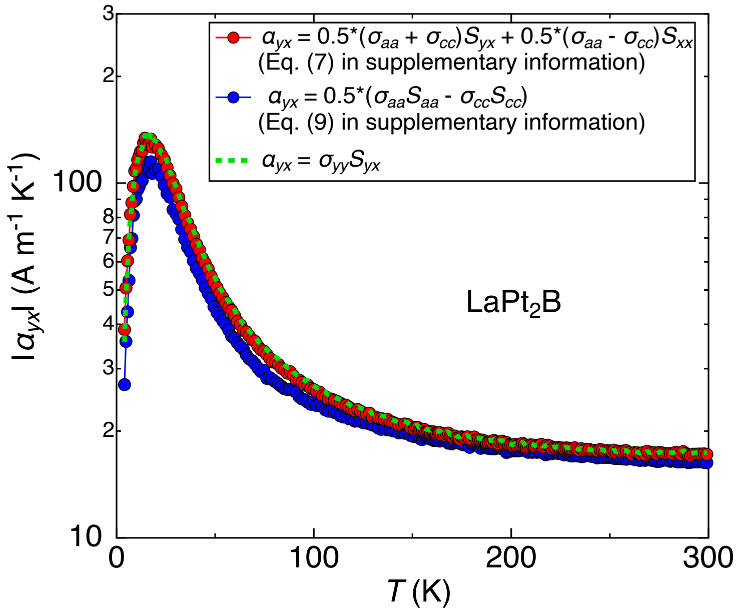

FIG. S12. **Evaluation of the Peltier conductivity.** Comparison of the Peltier conductivity in $LaPt_2B$ evaluated by several expressions.

temperature gradient ∇T. Then, θ_N is obtained from $\mathbf{E} = \hat{S}\nabla T$ as

$$\tan\theta_N = \frac{S_{yx}}{\sqrt{S_{xx}S_{yy}}} = \frac{S_{aa} - S_{cc}}{S_{aa} + S_{cc}}. \quad (12)$$

The Nernst angle represents the angle between the heat flow and the electric field, while the Peltier angle corresponds to the angle between the heat flow and the electric current.

J. Power factor

The multi-dimensional goniopolar conductor $LaPt_2B$ exhibits a giant Peltier conductivity α_{yx}, which results in high transverse thermoelectric performance. The transverse power factor (PF) is a measure of electrical power output for the transverse direction when a longitudinal temperature difference of 1 K is applied to the sample. Here we evaluated the transverse PF [9] of $LaPt_2B$ as

$$\mathrm{PF} = \frac{(S_{cc} - S_{aa})^2}{(\sqrt{\rho_{cc}} + \sqrt{\rho_{aa}})^2} \quad (13)$$

$$= \frac{4S_{yx}^2}{(\sqrt{\rho_{cc}} + \sqrt{\rho_{aa}})^2}. \quad (14)$$

Figures S13**a** and S13**b** show the comparison of the transverse PF of $LaPt_2B$ and several transverse thermoelectric systems. The transverse PF of $LaPt_2B$ exhibits a large value of 1.7 μW cm^{-1} K^{-2} at room temperature, which is considerably higher that that of known ANE-based systems.

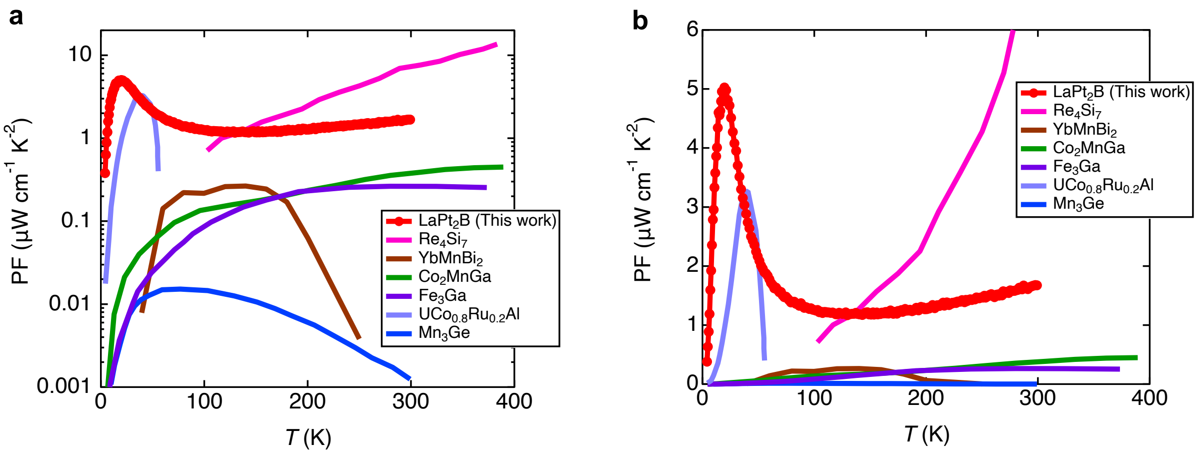

FIG. S13. **Transverse power factor. a,b**, Comparison of transverse power factors of several transverse thermoelectric systems on a semi-logarithmic scale (**a**) and a linear scale (**b**). The transverse thermoelectric materials shown in the figures are Re_4Si_7 [10], $YbMnBi_2$ [11], Co_2MnGa [12, 13], Fe_3Ga [14], $UCo_{0.8}Ru_{0.2}Al$ [15], and Mn_3Ge [16, 17].

K. Comparison of α_{yx} and θ_P when the heat-current is fixed at 45° froom crystallographic axes: $LaPt_2B$ and other transverse thermoelectric systems.

Here we evaluated the Peltier angle $\theta_P = \tan^{-1}(|\alpha_{yx}|/\sqrt{\alpha_{xx}\alpha_{yy}})$, the angle between the charge and heat currents as $\mathbf{J} = \hat{\alpha}(-\nabla T)$, for $LaPt_2B$ and other transverse thermoelectric systems, as shown in Fig. S14. In the present configuration where the heat current is applied at $\phi = 45°$ from in-plane direction, it yields $\theta_P = \tan^{-1}(|\sigma_{aa}S_{aa} - \sigma_{cc}S_{cc}|/|\sigma_{aa}S_{aa} + \sigma_{cc}S_{cc}|)$, and in goniopolar materials, this angle exceeds 45° since $|\sigma_{aa}S_{aa} - \sigma_{cc}S_{cc}| \geq |\sigma_{aa}S_{aa} + \sigma_{cc}S_{cc}|$. The transverse voltage disappears when the heat-current direction coincides with the crystallographic axis; therefore, the thermoelectric performance when the heat flow angle is fixed at 45° from the crystallographic axes is important in transverse thermoelectric materials. Note that the Peltier angle is slightly different from the Nernst angle of $\theta_N = \tan^{-1}(S_{yx}/\sqrt{S_{xx}S_{yy}})$, which expresses the angle between the electric field and heat current as $\mathbf{E} = \hat{S}\nabla T$. In realistic devices, the Peltier angle may have importance

since the electrical current is extracted under a certain temperature difference. In comparison

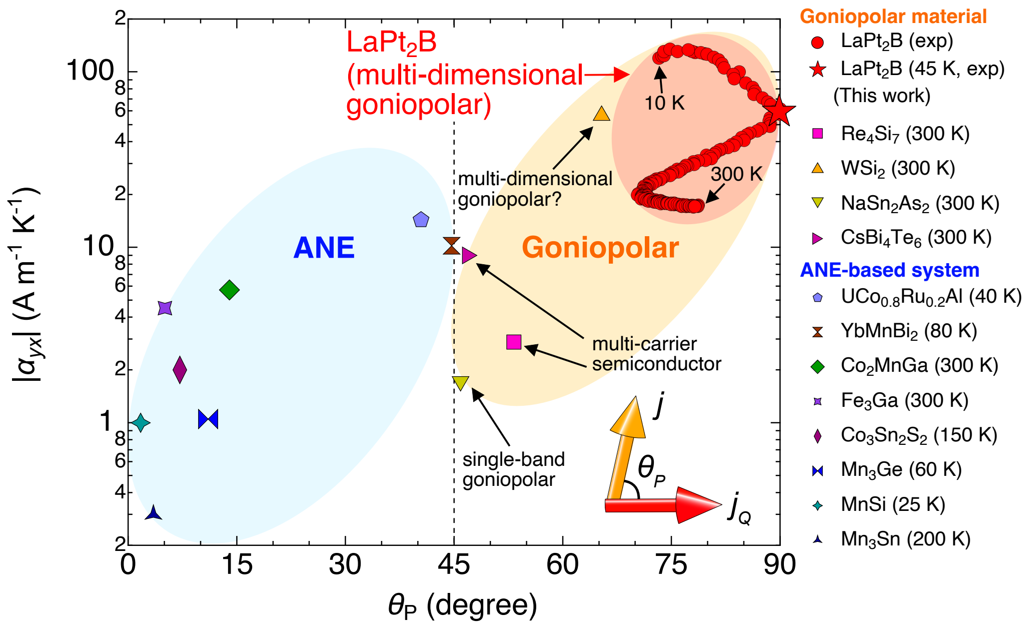

FIG. S14. **Peltier conductivity and Peltier angle.** Comparison of the Peltier angle θ_P of multi-dimensional goniopolar conductor $LaPt_2B$ with other transverse thermoelectric systems. In addition to the compounds shown in Fig. 5**a** in the main text, we added the data of MnSi [18], Mn_3Sn [19], WSi_2 [20], $NaSn_2As_2$ [21], and $CsBi_4Te_6$ [22]. Goniopolar systems exhibit thermopower with different polarities along the two crystallographic axes, which allows the Peltier angle exceeds 45 degrees. The inset illustrates the Peltier angle, which measures the angle between heat and charge current vectors.

to other goniopolar conductors and ANE-based systems, the magnitude of the transverse Peltier conductivity and Peltier angle in $LaPt_2B$ is remarkably prominent.

L. Evaluation of $z_{yx}T$

The dimensionless figure of merit $z_{yx}T$ of $LaPt_2B$ was evaluated using Eq. (3) in the main text. The $z_{yx}T$ of other ANE-based systems was evaluated as:

$$z_{yx}T = \frac{S_{yx}}{\rho_{yy}\kappa_{xx}}T. \tag{15}$$

Here, we consider the validity of the evaluation of $z_{yx}T$ in $LaPt_2B$. By directly measuring ρ_{yy} and κ_{xx}, we can also evaluate the $z_{yx}T$ of $LaPt_2B$ using Eq. (15). As shown in Fig. S15, the $z_{yx}T$

estimated using Eq. (15) exhibits slightly different behavior compared to the $z_{yx}T$ estimated using other expressions (the second and third terms in Eq. (3) in the manuscript). The difference in the estimation of $z_{yx}T$ may be attributed to measurement errors in κ_{xx}. Accurate measurement of κ_{xx} is difficult due to issues related to the sample's geometry.

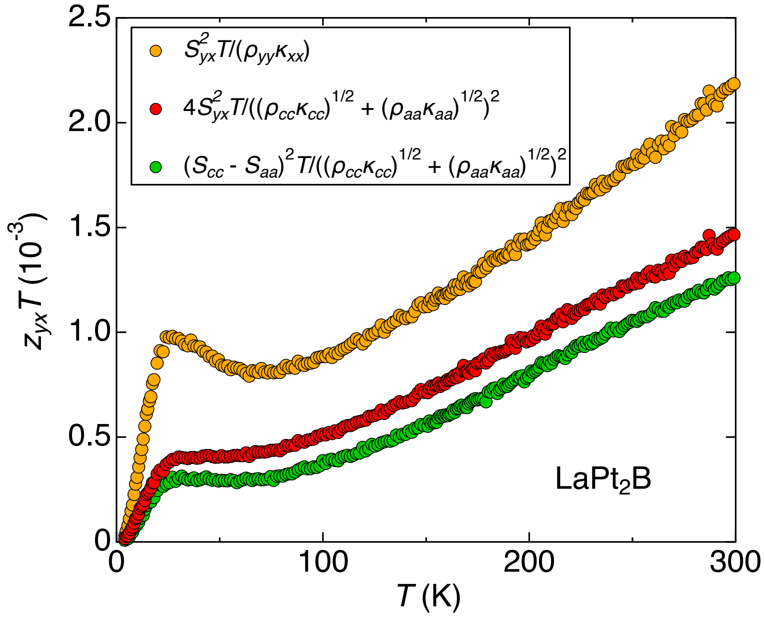

FIG. S15. **Evaluation of the dimensionless figure of merit for transverse thermoelectricity.** Comparison of $z_{yx}T$ in $LaPt_2B$ evaluated by several expressions.

[1] Chang, G., *et al.*, Topological quantum properties of chiral crystals. *Nat. Mater.* **17**, 978-985 (2018).

[2] Yao, T., *et al.*, First-principles study of structural stability, elastic and electronic properties of ternary rare earth-transition metal-Borides and carbides (RT_xZ, R = Sc, Y, and La, T= Pt and Pd, Z = B and C, and x = 2, 3, and 4). *Physica B* **431**, 58-68 (2013).

[3] Kawamura, M., FermiSurfer: Fermi-surface viewer providing multiple representation schemes. *Comp. Phys. Commun.* **239**, 197-203 (2019).

[4] Madsen, G. K. H., Carrete, J., & Verstraete, M. J., BoltzTraP2, a program for interpolating band structures and calculating semi-classical transport coefficients. *Comp. Phys. Commun.* **231**, 140-145 (2018).

[5] Giannozzi, P., Volja, D., Kozinsky, D., Fornari, M., & Marzari, N., Madsen, G. K. H., Carrete, J., & Verstraete, M. J., BoltzWann: A code for the evaluation of thermoelectric and electronic transport properties with a maximally-localized Wannier functions basis. *Comp. Phys. Commun.* **185**, 422-429 (2014).

[6] Lowhorn, N .D., Wong-Ng, W., Zhang, W., Lu, Z.Q., Otani, M., Thomas, E., Green, M., Tran, T. N., Dilley, N., Ghamaty, S., Elsner, N., Hogan, T., Downey, A. D., Jie, Q., Li, Q., Obara, H., Sharp, J., Caylor, C., Venkatasubramanian, R., Willigan, R., Yang, J., Martin, J., Nolas, G., Edwards, B., & Tritt, T., Round-robin measurements of two candidate materials for a Seebeck coefficient Standard Reference Material *Appl. Phys. A* **94**, 231-234 (2009).

[7] Sharma, G., Moore, C., Saha, S., & Tewari, S., Nernst effect in Dirac and inversion-asymmetric Weyl semimentals *Phys. Rev. B* **96**, 195119 (2017).

[8] Shao, Q., Kanakkithodi, A. M., Xia, Y., Chan, M. K. Y., & Grayson, M., Seebeck Tensor Analysis of (p × n)-Type Transverse Thermoelectric Materials *MRS Advances* **4**, 491-497 (2019).

[9] Zhou, C., Birner, S., Tang, Y,. Heinselman, K., & Grayson, M., Driving Perpendicular Heat Flow: (p × n)-Type Transverse Thermoelectrics for Microscale and Cryogenic Peltier Cooling *Phys. Rev. Lett.* **110**, 227701 (2013).

[10] Scudder, M. R., He, B., Wang, Y., Rai, A., Cahill, D. G., Windl, W., Heremans, J. P., & Goldberger, J. E., Highly efficient transverse thermoelectric devices with Re_4Si_7 crystals *Energy Environ. Sci.* **14**, 4009-4017 (2021).

[11] Pan, Y., Le, C., He, B., Watzman, S., J., and Yao, M., Gooth, J., Heremans, J., P., Sun, Y., & Felser,

C., Giant anomalous Nernst signal in the antiferromagnet $YbMnBi_2$ *Nat. Mater.* **21**, 203-209 (2022).

[12] Sakai, A., Mizuta, Y., P., Nugroho, A., A., Sihombing, R., Koretsune, T., Suzuki, M.-T., Takemori, N., Ishii, R., Nishio-Hamane, D., Arita, R., Goswami, P., & Nakatsuji, S., Giant anomalous Nernst effect and quantum-critical scaling in a ferromagnetic semimetal *Nat. Phys.* **14**, 1119-1124 (2018).

[13] Guin, S., N., Manna, K., Noky, J., Watzman, S., J., Fu, C., Kumar, N., Schnelle, W., Shekhar, C., Sun, Y., Gooth, J., & Felser, C., Anomalous Nernst effect beyond the magnetization scaling relation in the ferromagnetic Heusler compound Co_2MnGa *NPG Asia Materials* **11**, 16 (2019).

[14] Sakai, A., Minami, S., Koretsune, T., Chen, T., Higo, T., Wang, Y., Nomoto, T., Hirayama, M., Miwa, S., Nishio-Hamane, D., Ishii, F., Arita, R., & Nakatsuji, S., Iron-based binary ferromagnets for transverse thermoelectric conversion *Nature* **581**, 53-57 (2020).

[15] Asaba, T., Ivanov, V., Thomas, S., M., Savrasov, S., Y., Thompson, J., D., Bauer, E., D., Ronning, F., Colossal anomalous Nernst effect in a correlated noncentrosymmetric kagome ferromagnet *Sci. Adv.* **7**, eabf1467 (2021).

[16] Wuttke, C., Caglieris, F., Sykora, S., Scaravaggi, F. Wolter, A., U., B., Manna, K., Süss, V., Shekhar, C., Ferlser, C., Büchner, B., & H., Christian, Berry curvature unravelled by the anomalous Nernst effect in Mn_3Ge *Phys. Rev. B* **100**, 085111 (2019).

[17] Xu, L., Li, X., Lu, X., Collignon, L., Fu, X., Koo, J., Fauqué, B., Yan, B., Zhu, Z., Behnia, K., Finite-temperature violation of the anomalous transverse Wiedemann-Franz law *Sci. Adv.* **6**, eaaz3522 (2020).

[18] Hirokane, Y. *et al.*, Longitudinal and transverse thermoelectric transport in MnSi. *Phys. Rev. B* **93**, 014436 (2016).

[19] Ikhlas, M. *et al.*, Large anomalous Nernst effect at room temperature in a chiral antiferromagnet *Nat. Phys.* **13**, 1085 (2017).

[20] Koster, K. G. *et al.*, Axis-Dependent Conduction Polarity in WSi_2 Single Crystals. *Chem. Mater.* **35**, 4228-4234 (2023).

[21] He, B. *et al.*, The Fermi surface geometrical origin of axis-dependent conduction polarity in layered materials. *Nat. Mater.* **18**, 568-572 (2019).

[22] Chung, D.-Y. *et al.*, Anisotropy in Thermoelectric Properties of $CsBi_4Te_6$. *Mater. Res. Soc. Symp. Proc.* **793**, S6.1.1 (2004).
